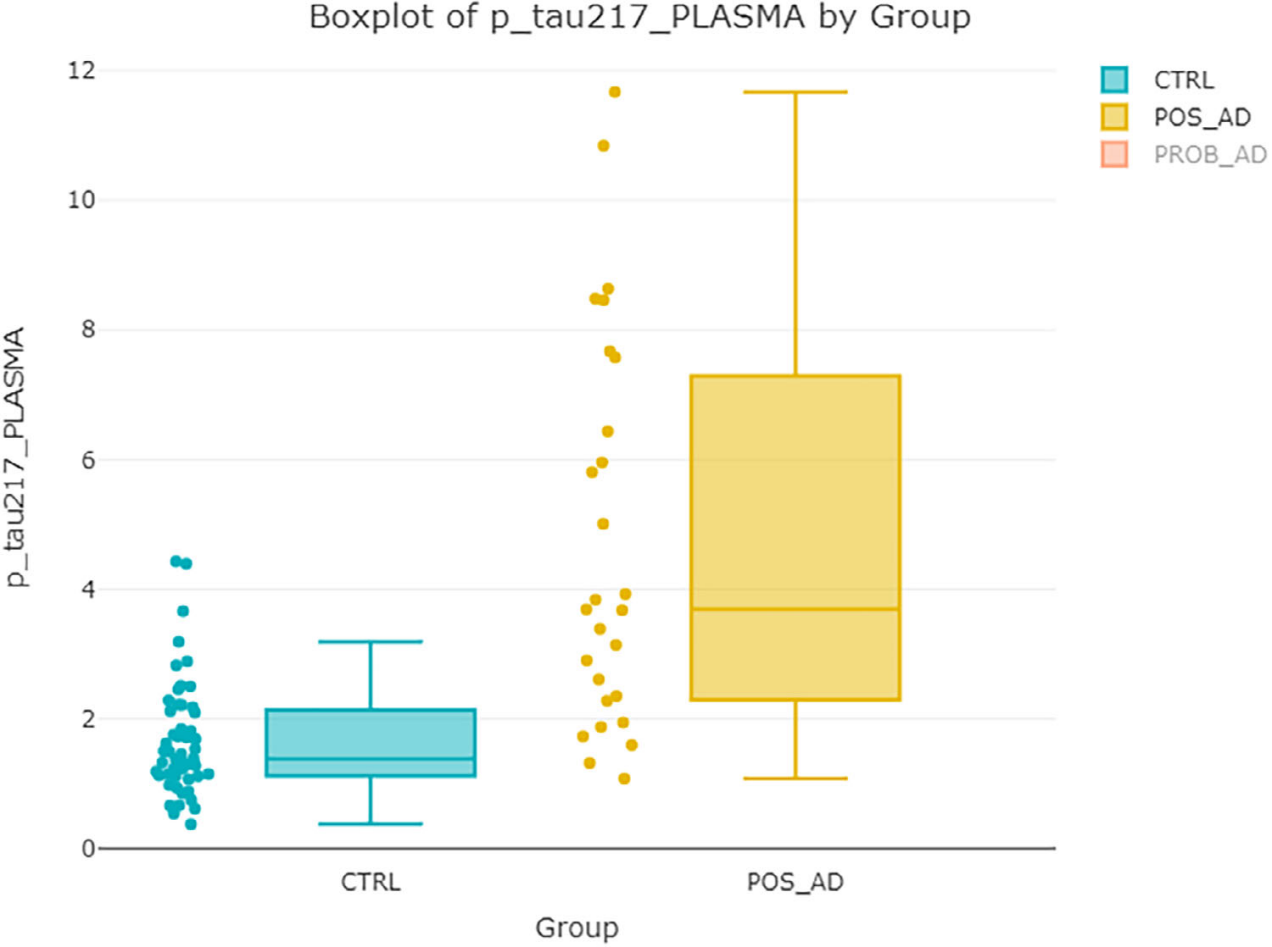# Different phosphorylated tau isoforms in biochemical diagnosis of Alzheimer’s disease

**DOI:** 10.1002/alz.090819

**Published:** 2025-01-09

**Authors:** Maciej Dulewicz, Przemyslaw Radoslaw Kac, Fernando Gonzalez‐Ortiz, Thomas K Karikari, Agnieszka Kulczynska‐Przybik, Barbara Mroszko, Michael Turton, Peter Harrison, Juan Manuel Maler, Timo Jan Oberstein, Johannes Kornhuber, Jörg Hanrieder, Henrik Zetterberg, Kaj Blennow, Piotr Lewczuk

**Affiliations:** ^1^ Department of Psychiatry and Neurochemistry, Institute of Neuroscience and Physiology, The Sahlgrenska Academy at the University of Gothenburg, Gothenburg, Gothenburg Sweden; ^2^ Department of Psychiatry and Neurochemistry, Institute of Neuroscience and Physiology, The Sahlgrenska Academy at the University of Gothenburg, Mölndal Sweden; ^3^ Institute of Neuroscience and Physiology, University of Gothenburg, Mölndal Sweden; ^4^ Institute of Neuroscience and Physiology, Department of Psychiatry and Neurochemistry, The Sahlgrenska Academy at University of Gothenburg, Mölndal, PA Sweden; ^5^ University of Pittsburgh, Pittsburgh, PA USA; ^6^ Department of Neurodegeneration Diagnostics, Medical University of Bialystok, Bialystok Poland; ^7^ Department of Neurodegeneration Diagnostics, Medical University of Białystok, Poland, Bialystok Poland; ^8^ Bioventix Plc, Farnham United Kingdom; ^9^ Department of Psychiatry and Psychotherapy, Universitätsklinikum Erlangen and Friedrich‐Alexander Universität Erlangen‐Nürnberg, Erlangen Germany; ^10^ University hospital Erlangen, Friedrich‐Alexander‐University Erlangen Nuremberg, Erlangen Germany; ^11^ Institute of Neuroscience and Physiology, Sahlgrenska Academy at the University of Gothenburg, Gothenburg Sweden; ^12^ Department of Neurodegenerative Disease, UCL Queen Square Institute of Neurology, University College London, London United Kingdom; ^13^ Hong Kong Center for Neurodegenerative Diseases, Hong Kong China; ^14^ Sahlgrenska University Hospital, Gothenburg Sweden; ^15^ Department of Neurodegenerative Disease, UCL Queen Square Institute of Neurology, University College London, London, ‐ United Kingdom; ^16^ Department of Psychiatry and Neurochemistry, Institute of Neuroscience and Physiology, The Sahlgrenska Academy, University of Gothenburg, Mölndal, Gothenburg Sweden; ^17^ Wisconsin Alzheimer's Disease Research Center, University of Wisconsin School of Medicine and Public Health, Madison, WI USA; ^18^ UK Dementia Research Institute at UCL, London United Kingdom; ^19^ Department of Psychiatry and Neurochemistry, Institute of Neuroscience and Physiology, The Sahlgrenska Academy, University of Gothenburg, Mölndal Sweden; ^20^ Clinical Neurochemistry Laboratory, Sahlgrenska University Hospital, Mölndal Sweden; ^21^ Department of Neurodegeneration Diagnostics, Medical University of Białystok, Białystok Poland

## Abstract

**Background:**

In the context of Alzheimer's disease (AD), blood‐based biomarkers have become increasingly important for various clinical purposes, such as screening patients and tracking the progression of the disease. Tau is a protein that stabilizes microtubules in nerve cells. In AD, different isoforms of tau become hyperphosphorylated, leading to the formation of neurofibrillary tangles, which are a key pathological feature of the AD. Measuring levels of different phosphorylated tau in the blood can provide insights into the extent of tau pathology in the brain. Phosphorylated tau (pTau) serves as a blood biomarker signaling the existence of AD‐related alterations, with pTau217, pTau181, and pTau231 proving to be significant indicators in this context. The primary goal of this study is to investigate the diagnostic utility of measuring plasma pTau217, pTau181 and pTau231 levels in identifying possible AD cases. This study intends to evaluate how effectively the concentrations of pTau217, pTau181 and pTau231 in the blood can differentiate individuals with CSF biomarker‐confirmed AD in comparison to CSF biomarker‐negative control group.

**Method:**

Plasma concentrations of pTau217, pTau181 and pTau231 were measured by in‐house Single molecule array (Simoa) assays developed at the University of Gothenburg (UGOT p‐tau217, pTau181 and pTau231). The quantitative assessment of classical biomarkers (Aβ‐42, Aβ‐42/Aβ‐40, tTau, and pTau181) in the CSF of patients with possible AD according to the Erlangen Score algorithm (ER 2 and 3) and controls (ER 0) were performed by Lumipulse.

**Result:**

Significantly The levels of pTau217, pTau181 and pTau231 correlated positively with CSF tTau, pTau181 and negatively with CSF Aβ1‐42 and Aβ ratio. The greatest effect size was observed for pTau217 (d=1.63), moderate for pTau181 (d=0.57) and pTau231 (d=0.3) respectively.

**Conclusion:**

The results of the present study indicate that plasma pTau217 could be the most valuable blood biomarker for AD diagnosis among the tested isoforms.